# Hot issues in triple-negative breast cancer

**DOI:** 10.20892/j.issn.2095-3941.2023.0294

**Published:** 2024-02-05

**Authors:** Xiaopeng Hao, Zefei Jiang

**Affiliations:** 1 Department of General Surgery, The First Medical Center of PLA General Hospital, Beijing 100036, China; 2 Department of Oncology Medicine, The Fifth Medical Center of PLA General Hospital, Beijing 100036, China

Triple-negative breast cancer (TNBC) is an aggressive disease with a poor prognosis. Several clinical trials have demonstrated future prospects for patients with TNBC based on improved long-term survival; however, there are still TNBC challenges, such as molecular classification and treatment optimization. Invited by *Cancer Biology & Medicine*, we would like to discuss four hot topics in TNBC and suggest some potential solutions (**Figure 1**).

**Figure 1:**
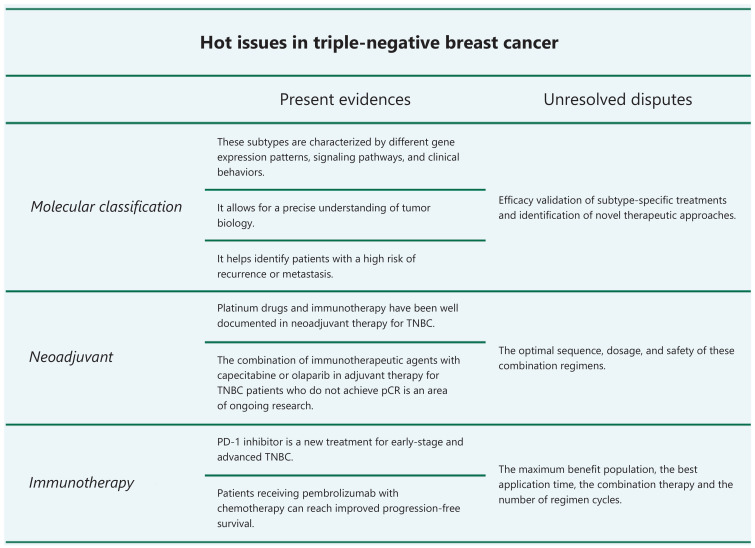
Hot issues in triple-negative breast cancer.

## What are the new advances in the molecular classification of TNBC and the significance for clinical practice?

TNBC is a refractory type of breast cancer that is characterized by high heterogeneity, which allows us to devise new methods of detection and identify additional targets to benefit patients. Molecular subtyping has revealed several distinct subtypes in TNBC, including basal-like 1 (BL1), basal-like 2 (BL2), immunomodulatory (IM), mesenchymal-like (M), and luminal androgen receptor (LAR) subtypes. These subtypes are characterized by different gene expression patterns, signaling pathways, and clinical behaviors. In general, the BL1 and BL2 subtypes are associated with high proliferative rates and aggressiveness, while the IM subtype displays immune-related gene expression patterns. Moreover, the M subtype exhibits mesenchymal features, and the LAR subtype is driven by androgen receptor signaling.

The treatment approaches for TNBC vary based on the molecular subtype. Chemotherapy remains the foundation of treatment for all subtypes, but targeted therapies and immunotherapies are emerging as potential options. Molecular subtyping of TNBC holds significant clinical implications. First, molecular subtyping allows for a precise understanding of tumor biology, which aids in tailoring treatment strategies. The LAR subtype may benefit from anti-androgen therapies, while the IM subtype may be sensitive to immunotherapies. Additionally, molecular subtyping helps identify patients at high risk for recurrence or metastasis, enabling personalized surveillance and treatment plans to improve the prognosis.

In conclusion, the molecular subtypes of TNBC, including BL1, BL2, IM, M, and LAR, provide valuable insight into tumor biology and have significant implications for clinical practice. The biological functions and molecular mechanisms underlying RNA binding protein abnormalities will also facilitate the discovery of new TNBC biomarkers and improve the therapeutic strategies^1^. Tailoring treatment strategies based on these subtypes may improve outcomes for TNBC patients. However, further research and clinical trials are urgently needed to validate the efficacy of subtype-specific treatments and identify novel therapeutic approaches to target different molecular subtypes of TNBC.

## What are the unresolved disputes involving TNBC neoadjuvant therapy, and how can the therapeutic strategies be further optimized?

Platinum-based chemotherapy, such as cisplatin or carboplatin, has been shown to be effective as a neoadjuvant treatment of TNBC^2^. These drugs damage DNA and impair tumor cell repair mechanisms. Platinum-based chemotherapy is often considered for TNBC patients with BRCA1/2 mutations or those with a high likelihood of a pathologic complete response (pCR). The utilization of platinum agents in combination with other chemotherapeutic drugs or targeted agents is an area of ongoing research to optimize treatment outcomes.

Immunotherapeutic agents, particularly immune checkpoint inhibitors (ICIs), such a pembrolizumab, have shown promise in the neoadjuvant treatment of TNBC^3^. These agents enhance the anti-tumor immune response by blocking immune checkpoint proteins, such as PD-1 or PD-L1. The ICIs are often used in combination with chemotherapy for neoadjuvant therapy. The addition of ICIs to chemotherapy, including platinum-based drugs or taxanes, increase pCR rates and improve prognosis; however, it is still unclear which chemotherapy drug is better in combination with immunotherapy drugs.

While immunotherapy has shown promise with respect to prognosis, immunotherapy is essential to manage and control immune-related adverse events (irAEs). Close monitoring, early recognition, and appropriate management of irAEs are crucial. Utilization of corticosteroids and other immunosuppressive therapies is helpful to control severe irAEs. Multidisciplinary collaboration between oncologists and immunologists is essential for the successful implementation of immunotherapy and the management of irAEs.

PARP inhibitors, such as olaparib or talazoparib, which target DNA repair pathways, have shown efficacy in TNBC patients with BRCA1/2 mutations. These agents can be used in neoadjuvant therapy as a targeted therapy to prevent repair of damaged tumor cell DNA. Clinical trials are ongoing to evaluate the efficacy of PARP inhibitors in combination with chemotherapy or immunotherapy to enhance treatment responses and improve outcomes in patients with TNBC.

Anthracycline-based chemotherapy, such as doxorubicin, is associated with cardiotoxicity and other adverse effects. The potential sparing of anthracycline-based drugs in neoadjuvant treatment of TNBC is an area of active investigation. While anthracyclines have traditionally been an essential component of neoadjuvant regimens, recent studies have explored alternative regimens without anthracyclines.

In conclusion, platinum drugs and immunotherapy have been well-documented as neoadjuvant therapy for TNBC and are recommended in the Chinese Clinical Society Breast Cancer Clinical Practice Guidelines. At present, however, it is necessary to further optimize the combination of immune and chemotherapy drugs. Moreover, the appropriate number of cycles need to be determined. Finally, adverse reactions need to be controlled.

## How can adjuvant therapy be optimized in TNBC patients with residual lesions after neoadjuvant treatment?

Capecitabine, an oral fluoropyrimidine, has been shown to be effective in the adjuvant treatment of TNBC patients who do not achieve a pCR after neoadjuvant treatment. Specifically, disease-free survival in this population was improved in clinical studies when capecitabine was incorporated into the adjuvant regimen. The decision to use capecitabine should be individualized when considering factors, such as patient tolerance, co-morbidities, and the overall risk-benefit ratio^4^.

Olaparib, a PARP inhibitor, benefits TNBC patients with germline BRCA1/2 mutations. For patients who do not achieve a pCR after neoadjuvant treatment and carry BRCA1/2 mutations, adjuvant olaparib can be considered to prevent repair of damaged tumor cell DNA^5^. The use of olaparib should be guided by genetic testing and individualized risk assessment.

The combination of immunotherapeutic agents with capecitabine or olaparib in adjuvant therapy for TNBC patients who do not achieve a pCR is an area of ongoing research. Preclinical and early clinical studies suggest potential synergistic effects between immunotherapies, such as ICIs, and chemotherapy or PARP inhibitors. These combinations enhance anti-tumor immune responses and improve prognosis. Additional clinical trials are warranted to determine the optimal sequence, dosage, and safety of these combination regimens.

In conclusion, optimizing adjuvant therapy for TNBC patients with residual lesions after neoadjuvant treatment involves individualized decision-making, patient tolerance, co-morbidities, and the overall risk-benefit ratio. Capecitabine and olaparib can be considered as treatment options based on the characteristics and biomarker status. The combination of immunotherapeutic agents with capecitabine or olaparib holds promise but requires further investigation to determine the most effective and safe approaches. Personalized adjuvant therapy strategies are intended to improve the prognosis in TNBC patients. Therefore, PD-L, circulating tumor DNA (ctDNA), tumor-infiltrating lymphocytes (TILS), and BRCA expression can be used to further classify the non-PCR population, thus enabling precise adjuvant intensive therapy.

## What progress has been made in immunotherapy for early and advanced TNBC?

Keynote 522 investigated the efficacy of immunotherapy in combination with chemotherapy in patients with early-stage TNBC^6^. The study evaluated the addition of pembrolizumab, an ICI, to neoadjuvant chemotherapy. The results showed significantly improved pCR and event-free survival rates in early-stage TNBC.

The cTRIO study was a multicenter, open-label phase II clinical study that included patients with stage II-III TNBC. The patients received 6 cycles of toripalimab (200 mg q3w) with nab-paclitaxel (125 mg/m^2^ on days 1 and 8 q3w) and carboplatin [area under the curve (AUC) = 2 on days 1 and 8 q3w]. The patients received 12 cycles of toripalimab after surgery. In this study 35 of 62 patients [56.5%, 95% confidence interval (CI): 43.3%–69.0%] achieved a pCR. Moreover, the pCR rate for lymph node-negative patients was 75%.

These clinical studies have laid the foundation for PD-1 inhibitors as a new treatment for early-stage TNBC. In advanced breast cancer, several clinical trials have promoted treatment progress. KEYNOTE-355 was a clinical trial that investigated the use of pembrolizumab in combination with chemotherapy as a first-line treatment for advanced TNBC^7^. The trial evaluated the addition of pembrolizumab to different chemotherapy regimens. The results demonstrated improved progression-free survival (PFS) in patients receiving pembrolizumab with chemotherapy, indicating the potential of this combination in advanced TNBC treatment. In the subgroup with a comprehensive positive score (CPS) ≥ 10, the objective response rate (ORR) of pembrolizumab was 52.7% and the median duration of response was 12.8 months. The ORR was 40.8% and the median duration was 7.3 months in the control group. The combination of pembrolizumab with chemotherapy for advanced TNBC is clearly beneficial.

TORCHLIGHT was a multicenter, randomized, double-blind, placebo-controlled phase III study that enrolled patients with advanced TNBC patients receiving first-line treatment. Compared to nab-paclitaxel, the combination of toripalimab significantly prolonged the PFS in patients expressing PD-L1, with a median PFS of 8.4 *vs.* 5.6 months (HR = 0.65, 95% CI: 0.47–0.91). In addition, a significantly increased trend in overall survival (OS) was also noted in the PD-L1-positive and intention-to-treat (ITT) populations in the toripalimab group. The median OS of the two groups of PD-L1-positive patients was 32.8 and 19.5 months, respectively (HR = 0.62, 95% CI: 0.41–0.91). These findings will create more treatment options for patients with advanced TNBC^8^.

Although immunotherapy in early and advanced TNBC has achieved some success in several clinical studies, there are still many challenges. Although immunotherapy can significantly improve the pCR and survival rates in patients with early-stage TNBC, the population that achieves maximum benefit, the best treatment timing, the superior combination therapy, and the number of cycles are still unknown. In patients with advanced TNBC it is hoped that immunotherapy will prolong survival and should be used as soon as possible; however, the currently approved treatment regimens only benefit PD-L1-positive TNBC patients. How to determine the best treatment combination and identify beneficial biomarkers remain challenges. In the future, how to further improve the efficacy of immunotherapy and utilize predictive markers are key to clinical application.
